# Deciding When to Align: Computational and Neural Mechanisms of Goal-Directed Social Alignment

**DOI:** 10.3390/brainsci15111200

**Published:** 2025-11-07

**Authors:** Aial Sobeh, Simone Shamay-Tsoory

**Affiliations:** 1 School of Psychological Sciences, University of Haifa, Haifa 3498838, Israel; sshamay@psy.haifa.ac.il; 2 Integrated Brain and Behavior Hub (IBBRC), University of Haifa, Haifa 3498838, Israel

**Keywords:** social decision-making, social alignment, mentalizing network, executive control network, goal-directed behavior

## Abstract

Human behavior is shaped by a pervasive motive to align with others, manifesting across a wide range of tendencies—from motor synchrony and emotional contagion to convergence in beliefs and choices. Existing accounts explain how alignment arises through predictive coding and observation–execution mechanisms, but they do not address how it is regulated in a manner that considers when alignment is adaptive and with whom it should occur. We propose a goal-directed model of social alignment that integrates computational and neural levels of analysis, to enhance our understanding of alignment as a context-sensitive decision process rather than a reflexive social tendency. Computationally, alignment is formalized as a prediction-error minimization process over the gap between self and other, augmented by a meta-learning layer in which the learning rate is adaptively tuned according to the inferred value of aligning versus maintaining independence. Assessments of the traits and mental states of self and other serve as key inputs to this regulatory function. Neurally, higher-order representations of these inputs are carried by the mentalizing network (dmPFC, TPJ), which exerts top-down control through the executive control network (dlPFC, rIFG) to enhance or inhibit alignment tendencies generated by observation–execution (mirror) circuitry. By reframing alignment as a form of social decision-making under uncertainty, the model specifies both the computations and neural circuits that integrate contextual cues to arbitrate when and with whom to align. It yields testable predictions across developmental, comparative, cognitive, and neurophysiological domains, and provides a unified framework for understanding the adaptive functions of social alignment, such as strategic social learning, as well as its maladaptive outcomes, including groupthink and false information cascades.

## 1. Introduction

Human behavior is profoundly shaped by a fundamental predisposition toward social alignment, defined as the coordination of behaviors or thoughts among group members [1]. Social alignment involves copying or matching another individual’s output—such as their choices, motor commands, or affective states—and manifests in a wide range of human tendencies, including motor synchrony, emotional contagion, and the dissemination of beliefs, preferences, and values [2]. This process underlies collective phenomena such as the coordination of mass movements and the formation of social norms [2].

Social alignment plays a critical role in preserving group unity, strengthening social bonds, and facilitating cultural transmission [3,4,5,6]. More recently, it has also been shown to enhance therapeutic alliance, prosocial behavior, parent–child bonding, and cooperation [7,8,9,10]. Despite its evident and widespread benefits, research over the past three decades has increasingly emphasized that alignment is not always adaptive [11,12,13,14]. The risks of alignment include groupthink, harmful peer pressure, and the propagation of false information cascades [1,15,16]. Imitating inappropriate gestures, adopting inaccurate beliefs, or conforming to majority choices when independent judgment is required can undermine both individual and collective outcomes. Accordingly, humans and animals more broadly may gain fitness advantages by aligning with others only to the extent that they adopt adaptive behaviors while avoiding maladaptive ones. Goal-directed selectivity in social alignment is therefore central to maximizing its benefits while minimizing its costs [17]. Indeed, theoretical and empirical studies show that selective alignment enhances the efficiency of social learning across human and non-human species [3].

Despite its ubiquity and importance, the mechanisms underlying goal-directed social alignment in humans remain underexplored. The advantages of goal-directed selectivity underscore the need for a regulatory decision system capable of strategically enhancing or inhibiting alignment. We propose that decisions about whether to align, and to what degree, involve mechanisms that integrate interpersonal and contextual cues to arbitrate between alignment and non-alignment. By combining insights from decision neuroscience with computational models of social decision-making, we extend existing accounts of automatic alignment to a goal-directed account that conceptualizes alignment as an adaptive decision process. Within this account, alignment decisions can be understood as deliberate choices between copying others and maintaining independence [18].

To situate our contribution, we first review current accounts of the computational and neural mechanisms underlying social alignment, with a particular focus on the neural circuitry that implements predictive coding processes facilitating automatic forms of alignment. While these accounts provide a foundation for understanding how individuals mimic behaviors, emotions, and thoughts or choices, they fall short of explaining how alignment is regulated in a context-sensitive manner. Building on this foundation, we propose an extended account that addresses not only how alignment occurs but also how the system decides when it should be expressed and with whom. We further discuss the empirical implications of our account for understanding both the adaptive and maladaptive facets of social alignment.

## 2. Current Mechanistic Accounts of Alignment

### 2.1. Computational Mechanism of Alignment: A Predictive Coding Account

Social alignment entails an update of one’s own output with those of others. The updating process can be captured in the neurocomputational framework of predictive coding [19]. According to predictive coding theory, the brain continuously forms expectations from prior experiences (top-down inputs) that predict incoming sensory events from the body and the external world [20]. A mismatch between these expectations and actual sensory signals (bottom-up inputs) constitutes a prediction error, signaling the occurrence of an unexpected event. Because unexpected events demand heightened attention and ongoing monitoring—metabolically costly processes—the brain is driven to minimize them to conserve energy. Thus, in response to prediction errors, the brain may either initiate actions to alter the incoming sensory information to match the predictions or adjust its internal model and prediction to match incoming data. Through this iterative process, prediction errors are progressively reduced over time [20].

Social alignment as a predictive coding process emphasizes adjustments in one’s own output to match that of others. This error minimization process can therefore be modeled using a reinforcement learning algorithm, in which prediction errors signal discrepancies between expected and observed social information and guide adaptive updating of beliefs or actions [21]. Here, the information that predictions are concerned with is the difference (or gap) between one’s own output and that of the other [19]. At any current time point, the gap is associated with an expected value *E_t_*(*G*), updated according to the discrepancy between the actual gap and the expected gap (Equation (1)) where *O_t_*(*G*) is the observed outcome (actual gap), *δ_t_* is the prediction error (observed minus expected gap), and *α* is the learning rate that dictates how much behavior is updated to reduce the observed discrepancy.

(1) $$E_{t+1}\left(G\right)=E_{t}\left(G\right)+\alpha \;\delta_{t},\;\delta_{t}=O_{t}(G)-\;E_{t}(G)$$

Complete alignment minimizes prediction errors, making the social interaction more predictable and less energy-consuming. It is thus proposed that during social interactions, a predictive coding mechanism is geared to maximize alignment in movement, emotion, and cognitions with others [2].

### 2.2. Neural Mechanism of Alignment

Consistent with the predictive coding account, Shamay-Tsoory et al. (2019) [2] proposed the ‘social alignment model’, in which alignment arises from a neural mechanism implementing iterative prediction–error minimization. This model involves a feedback loop comprising three interacting subsystems: (i) a gap detection system that monitors and signals prediction errors, (ii) an alignment system that generates behaviors aimed at minimizing those errors, and (iii) a reward system that reinforces prediction accuracy [2]. The misalignment detection system, anchored in the dorsal anterior cingulate cortex (dACC) and anterior insula (AI), monitors discrepancies between self and others, signaling prediction errors when an individual’s behavior, emotion, or cognition diverges from those of another [22,23,24]. (iii) The observation–execution (OE) system, encompassing the inferior frontal gyrus (IFG), inferior parietal lobule (IPL), superior temporal sulcus (STS), and premotor cortex, includes the mirror neuron system, which activates during both the observation and execution of behavior [25]. When observing another individual, the OE system maps their outputs (e.g., actions or emotional states) onto one’s own motor and perceptual representations, generating an impulse to match the observed output and thereby promoting alignment [26]. Finally, the reward system, including the ventral striatum (VS), ventromedial prefrontal cortex (vmPFC), and orbitofrontal cortex (OFC), reinforces alignment by rewarding the minimization of prediction errors associated with perceived gaps. This is evident in the intrinsic satisfaction linked to behavioral synchrony [27,28]. Together, these findings suggest that alignment is implemented by a neural architecture that detects gaps, generates congruent responses, and rewards successful matching of outputs.

## 3. Alignment Decisions: From Stimulus-Driven to Goal-Directed Alignment

The available neural and computational accounts described above conceptualize alignment primarily as an unregulated and stimulus-driven process. The computational account captures an automatic error-minimization mechanism that drives alignment, but it does not specify how the learning rate might vary adaptively according to context, thereby determining the degree of alignment. Similarly, the neural account describes a stimulus-driven process, whereby observing another person’s action recruits overlapping motor representations in the observer’s brain, predisposing them toward mimicry and shared outputs [29]. However, social alignment is often regulated, as observed in its selectivity which serves to maximize its benefits while preventing harmful consequences. It is therefore unlikely that alignment operates in an automatic and purely stimulus-driven manner [30]. 

Addressing this conceptual limitation requires reconceptualizing alignment as a regulated and goal-directed process. Formally, this requires extending the computational model to incorporate a hyperparameter-tuning function that allows the learning rate to vary as a function of contextual cues. It also requires extending the neural model to include monitoring and control neural mechanisms that regulate automatic alignment as a function of those informative contextual cues.

**Figure 1:**
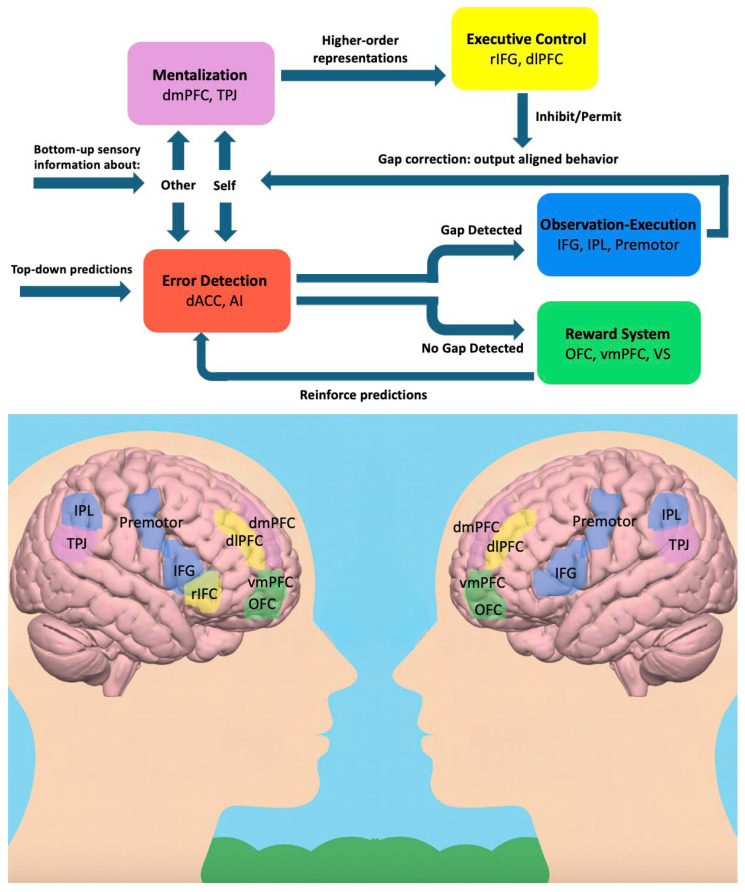
The five brain systems underlying goal-directed social alignment. Note: The model proposes that social alignment emerges from interactions among five large-scale brain systems. Bottom-up sensory information is processed in the gap detection system and the mentalizing system. The gap-detection system (red), comprising the dorsal anterior cingulate cortex (dACC) and anterior insula (AI), monitors the gap (or mismatch) between self- and other-generated sensory information, and it signals prediction errors when there is a discrepancy between expected and perceived gaps. The observation–execution system (blue), including the inferior frontal gyrus (IFG), inferior parietal lobule (IPL), and premotor cortex, generates alignment impulses by mapping others’ actions or states onto one’s own motor and perceptual representations. The reward system (green), comprising the orbitofrontal cortex (OFC), ventromedial prefrontal cortex (vmPFC), and ventral striatum (VS), reinforces successful alignment through prediction-error minimization. The mentalizing system (purple), including the dorsomedial prefrontal cortex (dmPFC) and temporoparietal junction (TPJ), represents the mental states, confidence, and expertise of others, providing higher-order contextual information about when alignment is likely to be adaptive. Finally, the executive control network (yellow), comprising the right inferior frontal gyrus (rIFG) and dorsolateral prefrontal cortex (dlPFC), implements top-down regulation by permitting or inhibiting alignment impulses based on these higher-order evaluations. Together, these interconnected systems enable alignment to operate as a goal-directed, context-sensitive decision process rather than an automatic reflex.

Here, we propose such a model, in which the alignment system operates in a goal-directed manner by incorporating regulatory control mechanisms that deliberately adjust the degree of alignment according to contextual cues and demands to achieve desired outcomes. In the proposed model, alignment is conceptualized as an innate tendency regulated via top-down control. Observation-Execution circuits generate automatic impulses to align, while higher-order monitoring and executive control networks evaluate contextual cues and selectively permit or suppress these impulses in a goal-directed fashion (Figure 1). Through the interaction of these neural components, the system can up-regulate alignment when convergence is adaptive, and down-regulate or inhibit it when adopting observed behaviors would be detrimental [31]. The following section outlines our proposed model in greater detail.

## 4. Proposed Account

### 4.1. The Computational Mechanism of Goal-Directed Alignment: Introducing a Meta-Learning Component

The computational mechanisms described in Section 2.1 conceptualize alignment as a process in which detecting a gap between self and other generates a prediction error, corrected proportionally to a learning rate. In standard formulations, this learning rate is treated as fixed, meaning that each given discrepancy between self and other is updated with equal percentage [21]. While this captures the general tendency for alignment, it does not address how the system regulates alignment, specifically, how it decides when and by how much to align and when to resist alignment.

A natural extension is to allow the learning rate to vary as a function of contextual variables [32,33]. In this formulation, the alignment system incorporates a meta-learning layer that adaptively tunes the size of updates. Specifically, the learning rate increases when the value of aligning with the other is deemed high, thereby accelerating convergence, and decreases when alignment is less valuable, thereby stabilizing independence. Formally:

(2) $$E_{t+1}\left(G\right)=\;E_{t}\left(G\right)+\;\alpha \;\delta_{t},\;\delta_{t}=\;O_{t}(G)-\;E_{t}(G)\;\alpha_{t}=\;f\left(Z_{t}\right)$$

where *Z_t_* represents a variable encoding the relative value of alignment versus nonalignment. This functional dependence transforms the learning rate from a static parameter into a context-sensitive control signal. In this way, alignment is no longer an inevitable consequence of prediction error correction but is regulated by higher-order assessments of whether aligning is likely to be adaptive in the current situation. But what are the variables that feed into this meta-learning function? What information does the brain rely on to decide when alignment should be strengthened and when it should be suppressed?

Extensive research in social psychology and behavioral ecology has identified numerous factors that influence alignment. Both fields converge on the finding that the decision to align is often strategic, deployed selectively to gain knowledge or social acceptance, and depends on the properties of the behavior being displayed, who is performing it, and whether the circumstances deem matching it beneficial [13,17,34]. For example, people are more likely to match another person’s choice if that choice appears to have a better payoff [3], if it is taken by someone perceived as reliable, expert, or familiar [34,35], and when one’s confidence in one’s own choice is low [36]. In other words, the decision to align is determined by various normative and informational motives that shape when, what, and whom individuals align with.

Among these variables, confidence emerges as a central input that regulates alignment. When driven by informational motives such as maximizing accuracy, deciding when, what, and whose choice to copy requires an estimate of confidence—the subjective probability that one’s own or the other’s choice is correct [34,37]. In computational models of reinforcement learning, confidence is well established as a signal that modulates the learning rate: low confidence increases updating, reflecting the need to learn rapidly under uncertainty, whereas high confidence decreases updating, reflecting stability when internal representations are reliable [38,39]. Within our framework, the learning rate indexes the degree of alignment, and relative confidence in self versus other may serve as a key regulatory variable tuning this learning rate, up-regulating alignment when self-confidence is low or partner-confidence is high, and down-regulating it when the opposite holds. Consistent with this view, numerous studies demonstrate that confidence critically determines the extent of social influence. When people feel highly confident in an initial judgment, they become less willing to revise their judgments or accept social advice [40]. Conversely, when individuals lack confidence in a choice, they are more likely to conform to others’ choice, and they are more inclined to request advice before finalizing their decision [41,42]. The person’s decision to align is influenced not only by their own confidence, but also by the confidence displayed by others. For example, advisors expressing their opinions with higher confidence exert greater sway over group decisions [43]. Together, these findings support the notion that relative self–other confidence governs the extent of social alignment. To formalize how this process might occur, the meta-learning function introduced in Equation (2) can be explicitly expressed as:

(3) $$\alpha_{t}=f(\frac{w_{0}\left(C_{Other,t}\right)}{w_{0}\left(C_{Other,t}\right)+\;w_{1}\left(C_{Other,t}\right)})$$

where *α* represents the context-sensitive learning rate at time *t*, and *C_self,t_* and *C_other,t_* represent the estimated confidence (or the subjectively estimated likelihood of accuracy) of self and other. The ratio between these confidence estimates defines the relative reliability of social versus personal information, such that a higher value increases the weight assigned to the other’s input. These confidence terms can be further modulated by perceived normative factors (represented in the weights *w* assigned to each term). The function describes a process in which the person integrates individual and social information in a Bayesian manner, to set a learning rate that flexibly adjusts the degree of alignment according to the inferred value of copying versus maintaining independence. Neurocomputational evidence supports this formulation. Bang et al. (2022) showed that confidence signals, both self- and other-related, are represented in distinct but interacting neural populations within the dorsomedial prefrontal cortex (dmPFC), which integrates these signals to guide the degree of behavioral adjustments that a person commits to match the behavior of the other person [44]. These results converge with behavioral evidence to suggest that confidence serves as a main computational variable feeding into the meta-learning function that governs learning rates in our model (Equation (2)), enabling selective, context-sensitive social alignment.

### 4.2. The Neural Mechanism of Goal-Directed Alignment

The neural account reviewed earlier describes how the interaction between the observation–execution system, the error-detection and reward circuits generates the impulses to align. While this account captures the stimulus-driven aspects of alignment, it does not capture the mechanisms that determine when and with whom to align—that is, how the brain selects when alignment is adaptive and when independence should be maintained. Deciding when and whom to copy often requires reasoning about others’ mental states—including their beliefs, intentions, and attitudes—and representing their traits, such as reliability, dominance, and expertise [34]. For example, estimating relative confidence in another person’s choice depends on the ability to infer that person’s uncertainty and to represent their credibility or expertise [44]. A large body of research situates these capacities within the mentalizing network, most prominently the dorsomedial prefrontal cortex (dmPFC) and temporoparietal junction (TPJ) [45,46].

The dmPFC and TPJ are consistently implicated in representing others’ traits and inferring their internal states, such as uncertainty in their decisions [45,46]. For example, Jiang et al. (2022) found that when participants judged the reliability of another person’s perceptual decisions, dmPFC selectively encoded the person’s decision uncertainty, whereas TPJ tracked the probability that the person’s choice was correct or incorrect based on their earlier performance [47]. Similarly, Bang and colleagues (2022) reported that dmPFC activity scaled with the inferred likelihood of error in a partner’s choice during social confidence judgments. These findings converge on the interpretation that dmPFC and TPJ track others’ likelihood of accuracy [44]. Furthermore, disrupting dmPFC with TMS impaired individuals’ ability to infer others’ intentions [48], and disrupting TPJ with tDCS impaired their perspective taking abilities [49]. Beyond its role in inferring mental states, the mentalizing network also contributes to evaluating others’ traits, such as dominance, social status, and expertise [50,51,52]. Boorman et al. (2013) demonstrated that as participants learned about advisors’ expertise by tracking the accuracy of their predictions, both dmPFC and TPJ encoded trial-by-trial updates in expertise assessment [52]. Together, these findings indicate that dmPFC and TPJ activity tracks trial-by-trial estimates of another person’s confidence, integrating information about their uncertainty and expertise. Specifically, TPJ provides estimates of whether others’ choices are likely to be correct, dmPFC represents their uncertainty, and both regions contribute to assessing their expertise.

Given its function, the mentalizing network is essential for determining whether alignment is likely to be adaptive. It likely provides the neural substrate for evaluating informational and normative factors relevant to alignment decisions, and by supplying these informative variables to the meta-learning mechanism, the mentalizing network helps regulate alignment, increasing or suppressing it according to contextual demands.

Activity within the mentalizing network is likely complemented by the executive control network (ECN), which supports shifting between independent decision-making and reliance on social information and enables the inhibition of automatic alignment impulses. The ECN is critical for cognitive flexibility, which is the capacity to switch between decision-making modes and strategies [53,54], and has been implicated in regulating transitions from automatic, stimulus-driven alignment to deliberate, mentalization-based alignment [30]. Functional connectivity studies show that coupling between ECN regions (e.g., the dorsolateral prefrontal cortex, dlPFC) and the mentalizing network increases when individuals decide whether to follow social advice or rely on personal experience [55]. In addition to supporting flexibility, the ECN plays a central role in inhibiting prepotent alignment behaviors, such as imitation and mimicry [56,57]. Neuropsychological studies demonstrate that lesions to ECN hubs, such as the right inferior frontal cortex, result in exaggerated imitative behavior [58,59]. Developmental evidence suggests that reciprocal connections between mentalizing and ECN regions enable adolescents to adaptively calibrate their reliance on social influence, thereby reducing susceptibility to harmful peer influence [60].

Together, the mentalizing network and the executive control network (ECN) provide complementary functions: while the dmPFC and TPJ represent others’ traits, accuracy, and confidence, the ECN supports the control mechanisms necessary to use these evaluations to regulate alignment (Figure 1). These two systems interact dynamically with the broader alignment circuitry described earlier. Through their coordinated activity, alignment can be upregulated when others are inferred to be highly confident, accurate, or expert, and downregulated when such cues are absent or misleading. This process may unfold temporally as follows: bottom-up sensory information about self- and other-generated behavior is first processed by both the gap-detection system (dACC, AI), which monitors discrepancies between expected and perceived alignment, and the mentalizing network (dmPFC, TPJ), which infers higher-order representations of others’ mental states and traits. When a discrepancy is detected, the observation–execution system (IFG, IPL, premotor cortex) generates automatic alignment impulses by mapping observed outputs onto one’s own representations. In parallel, the executive control network (rIFG, dlPFC) integrates the higher-order representations inferred by the mentalizing network and implements top-down regulation, either permitting or inhibiting alignment impulses generated by the observation–execution system. This sequence of bottom-up detection, contextual evaluation, and top-down control enables the network to dynamically arbitrate alignment in a goal-directed, context-sensitive manner.

## 5. Falsifiable Predictions and Empirical Implications

The proposed model yields several falsifiable predictions that can be empirically tested through behavioral, developmental, comparative, interindividual, and neurophysiological approaches. Developmentally, alignment is expected to be largely automatic early in life, and to become increasingly selective as prefrontal and executive functions mature. Supporting evidence shows that adolescents progressively integrate social information to reduce indiscriminate alignment as regions within the executive function network mature [60]. Comparatively, humans are predicted to demonstrate greater selectivity in alignment than non-human animals, reflecting the evolution of metacognitive mechanisms that enable assessment of one’s own and others’ confidence prior to aligning [34]. Behaviorally, selective alignment should deteriorate under conditions of cognitive load that tax the executive control network. Under load, individuals are expected to revert to more automatic imitation, showing increased alignment and reduced discrimination between reliable and unreliable partners. Interindividual differences in metacognitive and cognitive control functions are also expected to affect the efficiency of the proposed circuitry. For instance, individuals with overconfidence may assign excessive weight to self-confidence, leading to consistent under-alignment. Those with diminished cognitive flexibility may have a reduced capacity to switch between independence and alignment according to contextual demands. Similarly, metacognitive failures observed in certain clinical conditions, such as autism or schizophrenia, could distort confidence-based weighting, reducing the system’s ability to adaptively regulate alignment. Lastly, neurophysiologically, activity within the mentalizing network (dmPFC, TPJ) and executive control network (dlPFC, rIFG), as well as the functional connectivity between them, should predict behavioral selectivity in alignment tasks. Disrupting these regions through transcranial magnetic or direct current stimulation (TMS/tDCS) should impair regulatory control, resulting in heightened automatic alignment and diminished sensitivity to partner reliability. Collectively, these predictions offer a coherent empirical framework for testing the model.

## 6. Discussion and Broader Implications

Current neural and computational accounts of social alignment fall short of capturing its dynamic and context-sensitive nature. The model proposed fills this gap by introducing a goal-directed component to alignment. It formalizes alignment as an error minimization process equipped with a meta-control mechanism that adaptively tunes the learning rate according to the inferred value of aligning. Confidence, expertise, and reliability serve as key inputs to this regulatory function, represented neurally within the mentalizing network (dmPFC, TPJ). These evaluations interact with the executive control network (dlPFC, rIFG), which provides the top-down control necessary to permit or inhibit alignment impulses. Together, these systems transform alignment from an automatic reflex into a deliberate decision process that integrates social, cognitive, and contextual information.

Our model describes neurocognitive mechanisms that enable humans to optimize learning from social information, offering a mechanistic basis for psychological functions such as the control of imitation [61], adaptive empathy [62], and social influence on belief update [63]. It also provides a neural substrate for social learning strategies and collective intelligence. Furthermore, the model offers a novel perspective on the roots of maladaptive collective phenomena, such as groupthink and false information cascades, which we propose may arise from individual-level failures in the regulatory systems of alignment. Clinically and developmentally, disruptions in these goal-directed alignment mechanisms, particularly in the interaction between the mentalizing and executive control networks, may help explain social difficulties and rigid behavioral patterns observed during specific developmental stages and in conditions such as autism spectrum disorders and loneliness.

While offering a cohesive framework, the model also faces limitations that warrant further empirical and theoretical work. First, although we outline a plausible temporal sequence of interactions among the five neural systems, the precise temporal dynamics of their coordination remain to be tested with high-temporal-resolution methods. Second, the proposed meta-learning function specifies how self-versus-other confidence tunes the learning rate, but its formal structure requires empirical validation and likely further refinement. Third, the current formulation of the model primarily addresses informational influences on alignment, whereas various normative factors—such as perceived closeness, social pressure, or group identity—also likely exert additional control over alignment decisions and should be incorporated in future extensions of the model. Addressing these limitations will help further specify the model’s computational and neural architecture.

To conclude, by reframing alignment as a form of social decision-making under uncertainty, in which assessments of the states and traits of self and other serve as signals guiding whether to copy or maintain independence, this model shifts the focus from whether alignment occurs to when and how it should occur. It further delineates the neural architecture that enables these regulatory processes, thereby bridging automatic and goal-directed components of alignment. In doing so, it lays the groundwork for a unified framework that explains how alignment is adaptively controlled, illuminating the neural and computational principles that shape this fundamental social behavior.

## Data Availability

No new data were created or analyzed in this study. Data sharing is not applicable to this article.
